# Chitosan-*grafted*-poly(aniline-*co*-anthranilic acid) as a water soluble binder to form 3D structures for Si anodes†

**DOI:** 10.1039/c9ra10990k

**Published:** 2020-02-19

**Authors:** Eunsoo Kim, Rajeev K. K., Jaebin Nam, Junyoung Mun, Tae-Hyun Kim

**Affiliations:** Organic Material Synthesis Lab, Department of Chemistry, Incheon National University South Korea tkim@inu.ac.kr; Research Institute of Basic Sciences, Incheon National University 119 Academy-ro, Songdo-dong, Yeonsu-gu Incheon 406-772 South Korea; Department of Energy and Chemical Engineering, Incheon National University South Korea

## Abstract

We graft an electrically conductive poly(aniline-*co*-anthranilic acid) (PAAA) polymer capable of interacting with Si particles onto chitosan, a natural hydrophilic polymer, to form a chitosan-*grafted*-PAAA (CS-*g*-PAAA) copolymer, and use it as a new water soluble polymeric binder for Si anodes to relieve the physical stress resulting from Si volume change during charge/discharge cycles. The carboxylic acid functional groups within the PAAA structure, as well as the chitosan functional groups, bind to silicon particles to form a stable 3D network, resulting in high adhesion. Because the binder is conductive, the electrode using the CS-*g*-PAAA-8 : 1 with an optimal composition ratio of CS to PAAA of 8 : 1 shows a high initial capacity of 2785.6 mA h g^−1^, and maintains a high capacity of 1301.0 mA h g^−1^ after 300 cycles. We also extract chitosan directly from crab shells, and fabricate a Si@ECS-*g*-PAAA electrode by grafting PAAA onto the extracted-chitosan (ECS). This electrode records an initial capacity of 3057.3 mA h g^−1^, and maintains a high capacity of 1408.8 mA h g^−1^ with 51.4% retention after 300 cycles. Overall, we develop a polymeric binder with outstanding cell properties, ease of fabrication, and high water solubility for Si anodes by grafting a conductive PAAA onto chitosan.

## Abstract 2

We develop a polymeric binder with outstanding cell properties, and high water solubility for Si anodes by grafting a conductive PAAA onto chitosan.

## Graphical abstract

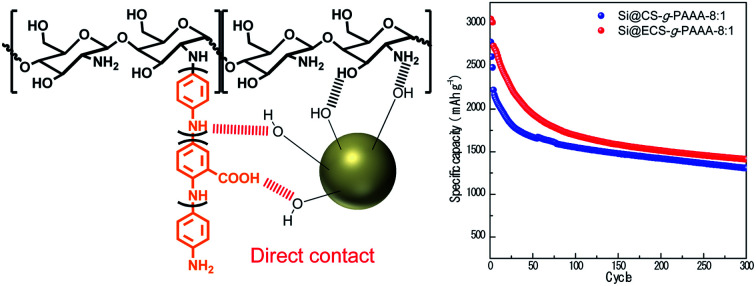

## 1. Introduction

Lithium ion batteries are in high demand for portable electronic devices, electric vehicles, and large-scale energy storage systems because of their high energy density, long cycle life, and light weight. Energy consumption patterns are also changing with the growing popularity of LIBs, and this has led to more extensive research on the development of high-capacity active materials for LIBs with enhanced energy density and durability.^1–3^

Si anodes have a theoretical capacity ten times higher than that of commercialized graphite anodes (approx. 4200 mA h g^−1^ for Si *versus* 372 mA h g^−1^ for graphite). Si also shows great promise as an active anode material with a potentially low electrochemical reactivity, low toxicity and relative abundance. Despite its high capacity, Si, however, undergoes a large volume change (up to 320%) in the lithiation/delithiation process, resulting in the pulverization and electrical isolation of silicon particles. This also forms an unstable solid electrolyte interface (SEI) layer on the Si surface, and the repeated charge/discharge process arising from the continuous use of Li^+^ ions causes the electrode to experience a rapid decline in capacity.^4–6^

For the past decade or so, many researchers have attempted to solve the problems caused by the volumetric expansion of Si anode materials. The different approaches include using Si/C composite materials to lower Si content and hence reduce the rapid decrease in capacity of Si/C electrodes,^7,8^ using nano-structured Si anodes such as Si nanowires,^9,10^ Si nanosheets^11^ and yolk-type Si nanoparticles,^12,13^ and alloying Si with other metals (Mg_2_Si, CaSi_2_, FeSi).^14–16^ These studies have improved the cycle stability of the cells using Si anodes, and reduced the pulverization caused by the rapid volume change of Si. However, direct modification of Si particles increases manufacturing costs, and the weaker contact with the Cu current collector contributes to the instability of the Si materials.

Taking a different approach, studies have also focused on developing polymeric binders. A binder adheres the active material and conducting agent to the current collector in the electrode. Poly(vinylidene difluoride) (PVdF) is popular as a binder for LIBs because of its outstanding chemical/thermal stability and electrolyte wettability, but cannot be used for Si anodes because its mechanical properties are not strong enough to suppress the volumetric expansion of Si.

Research has been conducted on binders capable of interacting with Si particles, such as poly(vinyl alcohol) (PVA), poly(acrylic acid) (PAA), and carboxymethyl cellulose (CMC). Functional groups such as hydroxyl (OH) and carboxylic acid (COOH) interact with Si particles and the Cu current collector, and the use of such polymers as binders has been reported to effectively improve the cycle capacity of Si electrodes.^17–25^ Despite strong adhesion to Si, the polymers' linear structure makes it difficult to effectively enclose Si particles, and they are thus less efficient in resolving the Si volume changes. Instead, binder materials that form 3D networks through crosslinking polymers, such as PAA–CMC^26^ and PAA–polyrotaxane^27^ with other polymers, have shown more effective adhesion to Si because they create a better contact. Polymeric binders with a 3D network structure are reported to have high tolerance to the structural changes arising from the volumetric expansion of Si, and are able to effectively suppress pulverization on the Si surface, thereby enhancing the capacity retention of the corresponding electrode.^26–33^

The use of electrically conductive binders for Si anodes has also been studied. These binders include poly(2,7-9,9-dioctylfluorene-*co*-2,7-9,9-(di(oxy-2,5,8-trioxadecane))fluorene-*co*-2,7-fluorenone-*co*-2,5-1-methylbenzoate ester) (PEFM),^34^ 3,6-poly(phenanthrenequinone) (PPQ)^35^ and poly(3,4-ethylenedioxythiophene):poly(styrene sulfonate) (PEDOT:PSS).^36^ When using a conductive polymer as a binder, direct contact between the conductive binders and Si can further lower resistance during charge transfer.^37–41^ However, these conductive polymers are typically soluble only in volatile organic solvents. Moreover, the synthesis and purification processes are complicated and expensive, and, most importantly, their poor interaction with Si particles limits their practical application as binders for Si.

We recently synthesized poly(aniline-*co*-anthranilic acid) (PAAA) by copolymerizing aniline and anthranilic acid, and used it as a binder for Si anodes. PAAA is not only easily prepared, but also contains carboxylic groups (COOH) that participate directly in hydrogen bonding with Si particles.^42^ The electrodes made of PAAA showed good adhesion strength and hence cycle performance up to 50 cycles. However, this polymer, PAAA, lacked eco-friendliness because it is sparingly soluble in water, making it difficult to fabricate the electrode, due to its very poor adhesion, in water. Therefore, it required the use of a toxic organic solvent like *N*-methyl-2-pyrrolidone (NMP) to fabricate the electrode. Furthermore, the PAAA-based electrode failed to show a long cycle life.^42^

In the present study, to preserve both good adhesion and the conductivity of the PAAA, we grafted PAAA onto the side chain of the chitosan main polymer, and used this chitosan-*grafted*-PAAA (CS-*g*-PAAA) as a water-soluble eco-friendly polymeric binder for Si anodes.

Chitosan (CS) is a natural polymer that has been drawing attention for various applications, including anti-microbial, water treatment, and wound healing. CS strongly adheres to Si due to its hydroxyl (–OH) and amine (–NH_2_) groups, and has been previously used as a binder material for Si.^43,44^ CS can also be easily obtained from the deacetylation of chitin, which is one of the most abundant natural polymers next to cellulose, and can also be easily extracted from crustaceans, crabs and shrimp.^45,46^ The abundance of amine groups in CS is also expected to facilitate the polymerization of aniline and anthranilic acid, making it compatible with PAAA.

**Fig. 1:**
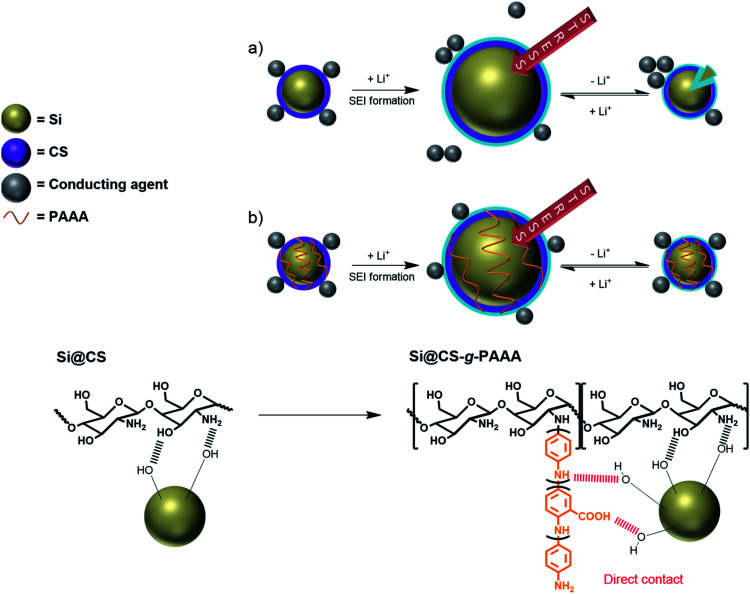
Schematic representation of the (a) liner contact formed by interaction between the chitosan and Si particles (Si@CS) and (b) 3D network formed by interactions between the chitosan-*grafted*-PAAA binder and Si particles (Si@CS-*g*-PAAA).

The chitosan-*grafted*-PAAA (CS-*g*-PAAA) binder, with chitosan as the main backbone and a PAAA side chain, has a 2D structure, but it can form a study 3D structure in the presence of Si particles (Fig. 1). This configuration allows the binder to control the volumetric expansion of Si more effectively, while suppressing the isolation of Si particles, and as a result, enhance the cycle stability of the cells.

## 2. Experimental

### 2.1. Materials

Chitosan (CS) was purchased from TCI, and aniline, anthranilic acid and ammonium persulfate (APS) were received from Sigma-Aldrich. Silicon nanoparticles (30–50 nm) were purchased from Alfa-Aesar. Hydrochloric acid and NaOH were purchased from Daejung. The electrolyte for all the experiments contained 1 M LiPF_6_ in combination with ethylene carbonate (EC) and ethyl methyl carbonate (EMC) (volume ratio of EC/EMC = 1/2 (v/v)) with 10% fluoroethylene carbonate (FEC), which was purchased from Panax Etec.

### 2.2. Preparation of chitosan from crab shells

#### Extraction of chitin from crab shell^46^

The crab shells (40 g) were washed several times with distilled water and dried in an oven at 40 °C for 48 hours. 3 g of the dried crab shells was made into a powder using a mortar, and this was dipped in 100 mL of 2 M HCl solution before heating at 80 °C for 24 hours to remove minerals and impurities. Next, proteins were removed by heating in 20 mL of 2 M NaOH solution. The filtrate was washed several times with acetone and distilled water, and dried in an oven at 40 °C for 48 hours. As a result, 7 g of white brown chitin was obtained. *ν*_max_ (ATR)/cm^−1^ 3440, 3260, 3100, 1656, 1620, 1556, 1376, 1308, 1072, 1012.

#### Deacetylation of extracted chitin to produce extracted chitosan^46^

3 g of extracted chitin was deacetylated by heating for 4 hours at 150 °C with 20 mL of 50% NaOH aqueous solution. The filtrate was washed with distilled water until a neutral pH was reached. Chitosan was obtained by drying in an oven at 40 °C for 48 hours (1.18 g). *ν*_max_ (ATR)/cm^−1^ 3352, 3292, 2864, 1656, 1592, 1420, 1376, 1320, 1060, 1024.

### 2.3. Synthesis of the poly(aniline-*co*-anthranilic acid) (PAAA)^42^

PAAA was synthesized according to our previously published methods. That is, after melting aniline (1.86 g, 20 mmol) and anthranilic acid (2.74 g, 20 mmol) in 1 M HCl 50 mL at room temperature, ammonium persulfate (11.3 g, 50 mmol) dissolved in 1 M HCl was slowly added, and synthesis was carried out with agitation under N_2_ at 5 °C for 24 hours (yield 3.08 g, 67%). *ν*_max_ (ATR)/cm^−1^ 3180, 3028, 1584, 1492, 1372, 1282, 1158.

### 2.4. Synthesis of the chitosan-*grafted*-poly(aniline-*co*-anthranilic acid) (CS-*g*-PAAA) and the extracted chitosan-*grafted*-poly(aniline-*co*-anthranilic acid) (ECS-*g*-PAAA)

#### CS-*g*-PAAA-8 : 1

1.00 g of chitosan was dispersed in 50 mL of 1 M HCl, completely dissolved by heating at 95 °C for 3 hours, and then cooled at room temperature. 125 mg (1.34 mmol) of aniline and 184 mg (1.34 mmol) of anthranilic acid monomers were added to the chitosan solution and agitated at 5 °C for 30 minutes. 766 mg (3.36 mmol) of ammonium persulfate was dissolved in a small amount of 1 M HCl solution, and a solution with dissolved chitosan, aniline and anthranilic acid was slowly added. This mixture was agitated under a N_2_ atmosphere at 5 °C for 24 hours. The solution was neutralized with NaOH (5 M, 20 mL) solution, and black products were acquired from ethanol deposition. Impurities were removed by washing several times with NMP and acetone followed by drying in a vacuum oven at 80 °C. The desired chitosan and PAAA copolymer (CS-*g*-PAAA-8 : 1) (0.9746 g, 74.6%) were obtained as a result. *ν*_max_ (ATR)/cm^−1^ 3286, 2864, 1584, 1490, 1430, 1374, 1322, 1152, 1066, 1030.

#### CS-*g*-PAAA-4 : 1

1.00 g of chitosan, 250 mg (2.68 mmol) of aniline, 368 mg (2.68 mmol) of anthranilic acid, and 1.53 g (6.71 mmol) of ammonium persulfate were used to produce the CS-*g*-PAAA-4 : 1 (1.22 g, 75.9%). *ν*_max_ (ATR)/cm^−1^ 3286, 2864, 1586, 1496, 1374, 1300, 1152, 1062, 1026.

#### ECS-*g*-PAAA-8 : 1

Synthesis was carried out using the same method as CS-*g*-PAAA-8 : 1. The extracted chitosan (0.60 g), and 75 mg (0.81 mmol) of aniline, 110 mg (0.81 mmol) of anthranilic acid, and 459 mg (2.01 mmol) of ammonium persulfate monomers were used to produce the ECS-*g*-PAAA-8 : 1 (429 mg, 55%).

### 2.5. Fabrication of the Si nanoparticle (SiNP) electrode

A homogeneous binder solution was obtained by agitating 60 mg of the polymeric binder in 1.2 mL of 1 M acetic acid solution at 80 °C for 3 hours. After dry mixing of 180 mg of silicon nanoparticles and 60 mg of Super P, the prepared binder solution was added to form an electrode slurry comprised of Si : Super P : binder in the ratio of 6 : 2 : 2 by weight. The slurry was cast on a Cu current collector with a doctor blade, and dried at 80 °C for 30 minutes. The electrode was then cut into a width of 1.13 cm^2^, and the remaining moisture was removed by vacuum drying at 120 °C for 6 hours. A coin cell was created in a glove box filled with argon (Ar). The electrodes fabricated from various binders were named Si@CS, Si@CS-*g*-PAAA-8 : 1, Si@CS-*g*-PAAA-4 : 1 and Si@ECS-*g*-PAAA-8 : 1.

Electrodes were fabricated with mass loading set at 0.6–0.7 mg cm^−2^ for Si@CS, Si@CS-*g*-PAAA electrodes of various compositions, and Si@ECS-*g*-PAAA electrodes fabricated from the extracted chitosan.

### 2.6. Characterization methods

XRD (X-ray diffractometer, Rigaku) with Cu K_α_ radiation (*λ* = 1.5412 Å) was carried out for the structural analysis of the silicon powder, and it was found to be the same as that of the typical Si nanoparticle (Fig. S1†).^47^

FT-IR spectra (Fourier-transform infrared spectroscopy, PerkinElmer Spectrum, two ATR spectrometer) were measured to examine changes in the functional groups of the synthesized polymer.

To measure the thermal stability of the polymer, a TGA (Thermogravimetric analysis, Scinco, TGA-N 150) instrument was used while increasing the temperature at 10 °C per minute from 30 °C to 700 °C under a N_2_ atmosphere. Changes to the chitosan under acidic conditions were examined by heating 100 mg of chitosan in 10 mL of 1 M HCl to 95 °C and agitating for 3 hours before dissolution. The solution was poured into an excess amount of acetone, and the resulting filtrate was prepared for TGA analysis by drying in a vacuum oven at 80 °C. TGA analysis was performed on various CS-*g*-PAAA and PAAA after vacuum drying.

To measure the mechanical properties of the polymer binder, a 3 M tape was placed onto the electrode, and a 180° peel-off test was conducted at 50 mm min^−1^ using a UTM.

Scanning electronic microscopy (JSM-7800F, JEOL) was employed to obtain images of the electrode surface.

### 2.7. Characterization of electrodes

A 2032-type coin cell (Wellcos) was used to measure cyclability and rate capability, and a three-electrode beaker cell was prepared in an argon-filled glove box to measure CV and EIS. The cell was assembled with a PE layer (Celgard 2400) as separator, and lithium metal as counter electrode. For the electrolytic solution, 1 M LiPF_6_ was dissolved in ethylene carbonate/ethylmethyl carbonate (EC : EMC = 1 : 2) containing 10% FEC additive. Lithium metal was the reference electrode during the CV and EIS measurements.

To measure cycle performance, a constant current test was conducted in the voltage range of 0.01–1.5 V *vs.* Li/Li^+^ using a CPS-Lab battery cycler (Basytec). The theoretical capacity of the electrode was determined by considering the weight ratio of Si, and formation was performed with the first three cycles at a low current of 0.1C. The current measured in the cycle after formation was 0.5C, and a discharge cycle was recorded.

To measure CV and EIS, tests were conducted using VSP (Bio-Logic) equipment. EIS was measured at an AC amplitude of 10 mV in the frequency range of 10 mHz to 100 kHz, and CV was obtained by scanning voltage at a rate of 0.1 mV s^−1^.

### 2.8. Solubility test

50 mg of chitosan and CS-*g*-PAAA-8 : 1 polymers were each dissolved in 1.0 mL of 1 M acetic acid by heating and agitation at 80 °C for 3 hours, and the result was inspected with the naked eye.

## 3. Results and discussion

### 3.1. Synthesis and characterization

#### Chitosan-*grafted*-poly(aniline-*co*-anthranilic acid) (CS-*g*-PAAA)

Aniline and anthranilic acid were grafted onto chitosan by oxidative polymerization using ammonium per sulfate (APS) as an initiator to produce chitosan-*grafted*-poly(aniline-*co*-anthranilic acid) (CS-*g*-PAAA) binders with various PAAA compositions. The polymeric binders were designed to provide both strong adhesion to Si and electrical conductivity (Scheme S1†). CS-*g*-PAAA copolymers with various PAAA compositions were prepared by changing the amount of anthranilic acid and aniline, while the CS content was fixed. Copolymers with a CS : PAAA mass ratio of 8 : 1 and 4 : 1 were prepared, and were designated CS-*g*-PAAA-8 : 1 and CS-*g*-PAAA-4 : 1, respectively. The ratio of anthranilic acid to aniline was set to 1 : 1 for both samples.

To avoid the possibility of blending, the product (CS-*g*-PAAA) was washed several times with NMP (which can dissolve PAAA well) to remove all PAAA and any impurities that are not graft-polymerized onto chitosan before proceeding with FT-IR measurement and electrode fabrication.

Comparative spectroscopic analysis using FT-IR was carried out to confirm the structure of the chitosan-*grafted*-poly(aniline-*co*-anthranilic acid) (CS-*g*-PAAA) (Fig. S2a and Table S1 in ESI†). The characteristic peaks of chitosan (the amide C=O stretching at 1654 cm^−1^, the primary amine NH_2_ bending at 1592 cm^−1^, and the C–O stretching both at 1058 and 1026 cm^−1^) and PAAA (the quinoid C=C stretching at 1574 cm^−1^, the benzenoid C=C stretching at 1492 cm^−1^, the aromatic C–H bending at 1140 cm^−1^) were all observed. In addition, the relative integrals of the benzenoid peak (at 1492–1506 cm^−1^) of PAAA to the peak corresponding to the C–O stretching of CS (at 1026 cm^−1^) were calculated, to determine the compositions of CS-*g*-PAAA.^48,49^ The other peaks overlapped one another, and thus could not be used for quantitative analysis. The relative integrals of the peaks for the benzenoid C=C to alcohol C–O increased as the feed ratio of anthranilic acid + aniline increased, confirming the increased compositions.

**Fig. 2:**
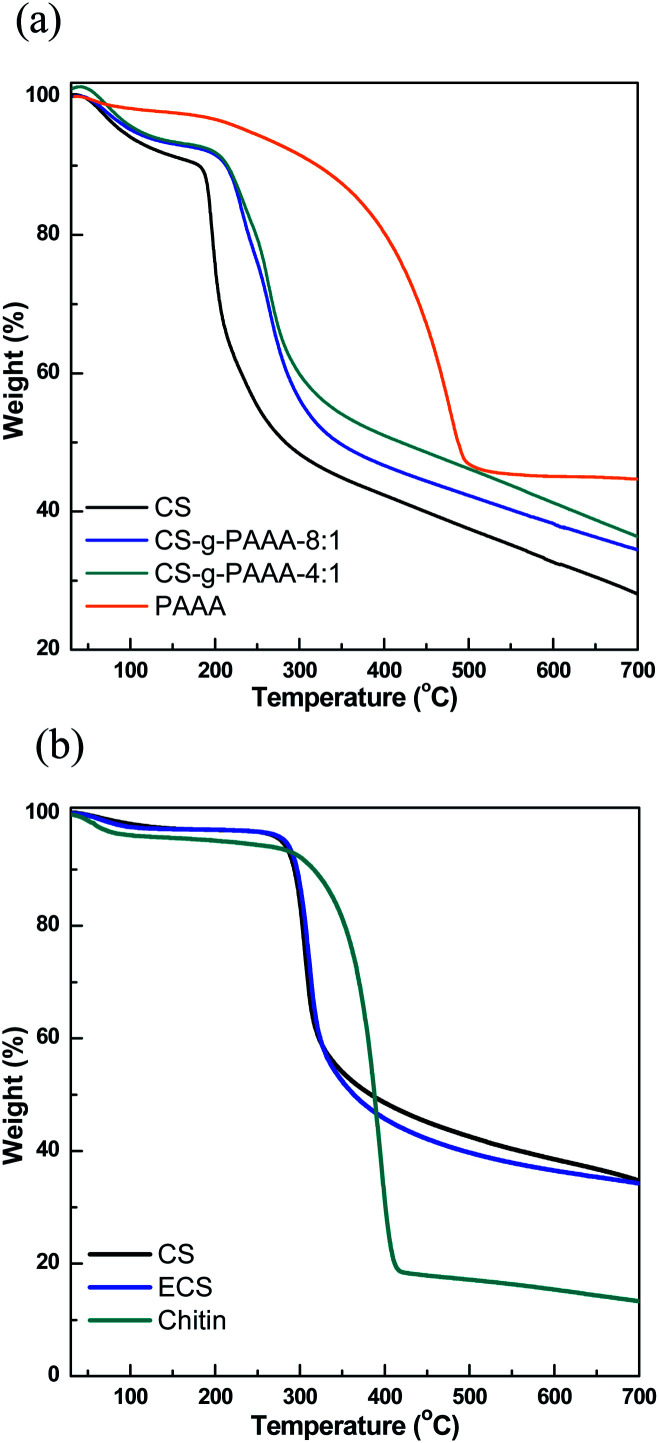
TGA graphs of (a) CS, CS-*g*-PAAA-8 : 1, CS-*g*-PAAA-4 : 1 and PAAA, and (b) commercially available CS, chitosan from crab shell and chitin extracted from crab shell.

The different compositions of the CS-*g*-PAAA copolymers were further examined using thermal gravimetric analysis (TGA). A greater % weight loss was recorded in the range of 180 °C to 320 °C for chitosan with a smaller amount of anthranilic acid + aniline, further supporting a higher composition of chitosan (Fig. 2 and Table S2†).^48,49^ No meaningful differences were observed from experiments other than FT-IR and TGA analyses, possibly because the amount of grafted PAAA is not high compared to CS.

#### Extracted chitosan

**Fig. 3:**
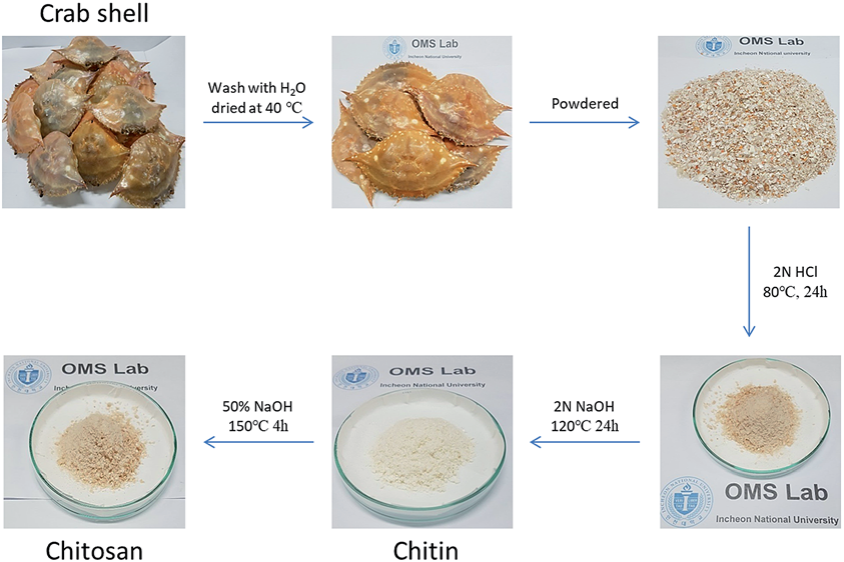
Procedure for chitosan extraction from crab shell.

We also grafted PAAA onto chitosan that had been extracted from crab shells, to produce extracted chitosan-*grafted*-PAAA (ESC-*g*-PAAA), following the same procedure used to graft PAAA onto the commercially available CS (CS-*g*-PAAA). The extracted chitosan (ECS) was first obtained by deacetylation of chitin, which was obtained from crab shells (Fig. 3).

The extracted chitosan was characterized using FT-IR spectroscopy (Fig. S2b†). Typical peaks of the primary amide (at 1656 and 1620 cm^−1^) and secondary amide (at 1556 cm^−1^) were observed. For the primary amide, the peak at 1656 cm^−1^ was due to carbonyl, and the hydrogen bond with N–H, and the peak at 1620 cm^−1^ was due to C=O and interactions with the hydroxyl groups in the molecules. The peaks decreased with deacetylation, and a new NH_2_ bending peak (at 1592 cm^−1^) indicated that chitosan was successfully extracted.^49^ Compared to commercially available chitosan, the intensity of the NH_2_ peak was more prominent, while all other peaks were identical, suggesting that the degree of deacetylation was somewhat higher for the extracted chitosan (ECS). This was also evident in the subsequent adhesion strength and cyclability to some extent (see below).

### 3.2. Mechanical properties of the Si@CS-*g*-PAAA and Si@ECS-*g*-PAAA electrodes

**Fig. 4:**
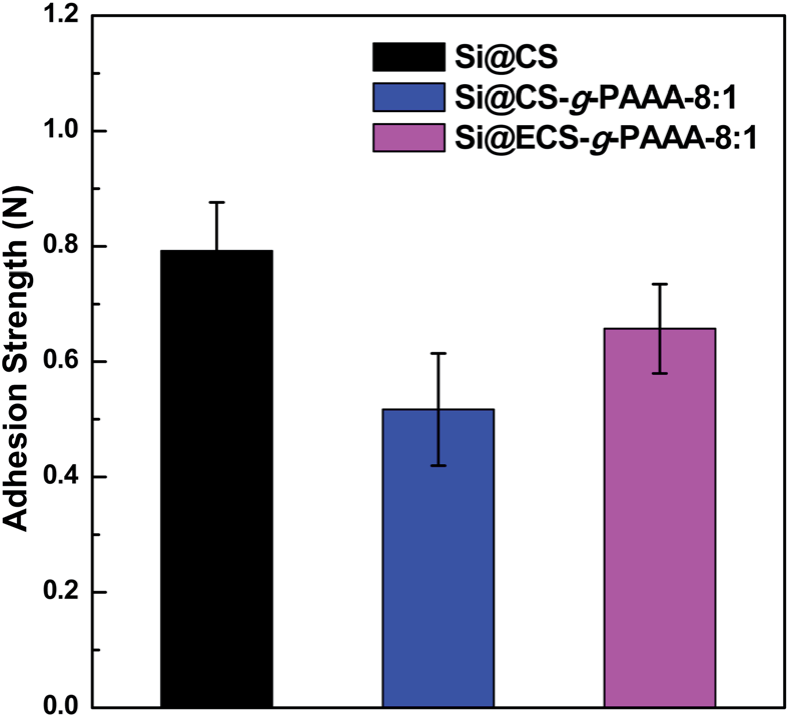
Adhesion strengths of Si@CS, Si@CS-*g*-PAAA-8 : 1 and Si@ECS-*g*-PAAA-8 : 1.

To examine the mechanical properties of the synthesized CS-*g*-PAAA polymer, a 180° peel off test was conducted on Si electrodes (Si@CS-*g*-PAAA) fabricated using CS-*g*-PAAA with various PAAA compositions, and a mass ratio of 6 : 2 : 2 (active material : binder :  conducting agent). The results were compared to those of a CS electrode (Si@CS) fabricated in the same ratio, using a pristine chitosan binder (Fig. 4).

The chitosan electrode showed the strongest adhesion, while the Si@CS-*g*-PAAA-4 : 1 electrode with the most PAAA (that is, the least amount of chitosan) exhibited an adhesion strength that was too weak to be measured. Increasing the amount of PAAA weakened the adhesion strength (Si@CS-*g*-PAAA-4 : 1 < Si@CS-*g*-PAAA-8 : 1 < Si@CS) (Fig. 4). This can be ascribed to the numerous hydroxyl (–OH) and amine (–NH_2_) groups in chitosan interacting with the hydroxyl groups on the Si surface.

The adhesion strength of the Si@ECS-*g*-PAAA binder, which was prepared by grafting PAAA onto extracted chitosan, was also measured under the same conditions. The ratio of extracted chitosan (ECS) to PAAA was set to 8 : 1 throughout this study because the electrode prepared from the Si@CS-*g*-PAAA-8 : 1 showed the best cyclability and coulombic efficiency (this will be discussed later). The Si@ECS-*g*-PAAA-8 : 1 electrode, prepared from the extracted chitosan, displayed somewhat higher adhesion than the Si@CS-*g*-PAAA-8 : 1 electrode with the same CS : PAAA ratio, and again this can be ascribed to the presence of slightly higher amounts of amine and amide groups in the ECS (Fig. 4).

### 3.3. Evaluation of electrochemical cell performance

**Fig. 5:**
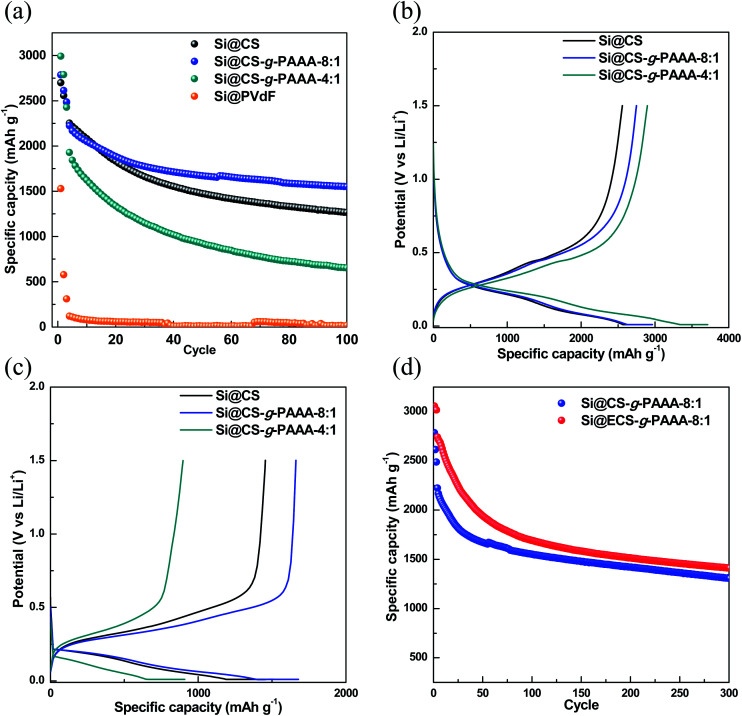
(a) Specific capacity, (b) voltage profiles of the formation cycle (0.1C), (c) voltage profiles at 50 cycles (0.5C) of the Si@CS (black circle), Si@CS-*g*-PAAA-8 : 1 (blue circle), Si@CS-*g*-PAAA-4 : 1 (dark cyan circle) and Si@PVdF (orange circle) electrodes, and (d) specific capacity of Si@CS-*g*-PAAA-8 : 1 (blue circle) and Si@ECS-*g*-PAAA-8 : 1 (red circle).

In order to examine the effect of PAAA, a conductive polymer reported in our previous paper,^42^ when it is grafted to CS, the cell performance of Si electrodes fabricated using the CS-*g*-PAAA copolymer binder was evaluated, and compared to an electrode with CS and PVdF binder as a reference. A charge/discharge cycle test was conducted at a low current (0.1C) for the first three cycles, and then the discharge capacity was measured at 0.5C for the following cycle (Fig. 5a).

First of all, the Si@PVdF electrode showed a very low initial capacity of 1528 mA h g^−1^ and also showed a very low capacity of 13.4 mA h g^−1^ at 100^th^ cycles, indicating that the PVdF binder is not applicable to the Si electrode due to its very low interaction with Si. No further data were, therefore, included.

Next, initial capacity of the electrode made with only chitosan (Si@CS) as binder was 2670 mA h g^−1^, and the capacity after 100 cycles was 1253.4 mA h g^−1^, showing a overall capacity retention of 55.6%. In contrast, the Si@CS-*g*-PAAA-8 : 1 electrode showed an initial capacity of 2785 mA h g^−1^ and capacity of 1546.0 mA h g^−1^ after 100 cycles, showing the highest capacity retention of 69.5% among the electrodes measured.

The Si@CS-*g*-PAAA-4 : 1 electrode, which had the highest content of PAAA, showed the highest initial capacity of 2992 mA h g^−1^ among the three electrodes prepared under the same mass loading, but its capacity dropped to 643 mA h g^−1^ after 100 cycles, thereby exhibiting the lowest capacity retention of only 33.4%.

To summarize this performance in more detail, the initial capacity of the electrodes increased with the amount of PAAA (Fig. 5a and b) because a greater amount of silicon was activated, with an effective transfer of electrons to silicon, due to the direct contact between the electrically conductive PAAA binder and Si surface. Therefore, the Si@CS-*g*-PAAA-4 : 1 electrode with the highest PAAA content showed the highest initial discharge capacity. However, this electrode experienced a rapid decrease in capacity after the formation cycles, translating to a poor retention capacity. As confirmed by the peel-off test, the excess PAAA caused the Si@CS-*g*-PAAA-4 : 1 electrode to have weaker adhesion to the Cu foil and Si, and consequently Si particles were detached from the current collector, causing an increase in electrical isolation. As a result, the active material (Si) was unable to retain its originally high capacity during the charge/discharge cycles.

The Si@CS-*g*-PAAA-8 : 1 electrode, on the other hand, showed better capacity retention than all the other electrodes, and was even higher than the Si@CS electrode. This is ascribed to the strong contact between this binder and Si.^40,50,51^ This resulted from the formation of the 3D structure between the PAAA unit in the CS-*g*-PAAA-8 : 1, in combination with the hydroxyl and amine groups in CS, and Si (Fig. 1).

The initial coulombic efficiency (ICE) and coulombic efficiency (CE) of the two Si@CS-*g*-PAAA and Si@CS electrodes were then compared. The Si@CS-*g*-PAAA-8 : 1 electrode again showed the highest ICE value (63.9% (for Si@CS); 69.0% (for Si@CS-*g*-PAAA-8 : 1) and 64.1% (for Si@CS-*g*-PAAA-4 : 1)) (Fig. 5b). In general, CE is closely related to the formation of the SEI layer, and the low CE in the first cycle was caused by the irreversible formation of SEI, which is a factor that lowers ICE. When the binder with the best cell performance (CS-*g*-PAAA-8 : 1) was used, direct physical contact was achieved between the conductive polymeric binder and silicon. This facilitated the transfer of electrons and increased ICE, thereby forming an SEI layer of appropriate thickness. Accordingly, we can expect a decrease in the resistance of SEI (to be further discussed).

The CE values at 100 cycles were measured to be 98.6% for Si@CS, 99.4% for Si@CS-*g*-PAAA-8 : 1 and 99.0% for Si@CS-*g*-PAAA-4 : 1. The CS-*g*-PAAA electrode achieved a higher CE than that of the CS electrode because the CS-*g*-PAAA binder forms a stable 3D network with Si, thus effectively suppressing the volumetric expansion of Si. Accordingly, the reduced isolation of Si and SEI formation during volumetric expansion and contraction in the charge/discharge process suppresses the current loss caused by additional SEI formation. That is, the effective suppression of volumetric expansion in the electrodes with high CE inhibits the isolation of Si, caused by the formation of the stable SEI layer, which was then induced by the 3D network which was formed between the binder and Si.

The cycle data of the Si@CS-*g*-PAAA-4 : 1 electrode, which contained the least amount of CS, showed a lower CE than that of the Si@CS-*g*-PAAA-8 : 1 electrode. This is because the SEI layer could not be properly maintained given the poor adhesion of the Si@CS-*g*-PAAA-4 : 1 electrode. As a consequence, the current was used to form a new SEI layer, causing a reduced CE. These results suggest that the introduction of PAAA to CS was effective as an Si binder, but the poor adhesion of the binder in the presence of excess PAAA reduced CE and the retention capacity of the electrode. This made the cycle results worse than those of the pristine chitosan electrode (Si@CS).

Moreover, from the voltage profile during the formation cycle of the electrodes (Fig. 5b), we did not observe any special behavior in the voltage range of 0.01–1.5 V *vs.* Li/Li^+^, suggesting that the Si charge/discharge process was not affected by CS and the CS-*g*-PAAA polymers. The capacity of the Si@CS-*g*-PAAA-8 : 1 electrode after 50 cycles was higher than electrodes with other binders (Si@CS and Si@CS-*g*-PAAA-4 : 1). This is consistent with the small charge–discharge potential difference of the Si@CS-*g*-PAAA-8 : 1 electrode shown in the voltage profile, indicating a low polarization of this electrode (Fig. 5c). Since the CS binder in the Si@CS electrode is not conductive, there is no direct conductive path on the surface of the Si active materials. The conductive CS-*g*-PAAA-8 : 1 binder, on the other hand, had direct contact with Si, and hence polarization was reduced due to low resistance during charge/discharge.

In addition, the rate performance of the three electrodes using different binders (Si@CS, Si@CS-*g*-PAAA-8 : 1 and Si@CS-*g*-PAAA-4 : 1) were evaluated at different current densities of 0.1C, 0.5C, 1C, 2C and 5C for running 3 cycles for discharge and restored at 0.1C (Fig. S3†). As with the cycle performance data, the addition of PAAA resulted in the high capacity maintained for the Si@CS-*g*-PAAA-8 : 1 electrode, and the Si@CS-*g*-PAAA-4 : 1 electrode having a higher content of PAAA showed a significantly lower capacity at 5C than those of the CS and CS-*g*-PAAA-8 : 1 due to its low adhesion property.

Overall, the following conclusions can be derived from the above electrochemical results. Firstly, the strong adhesion of CS keeps the Cu current collector and active materials (Si) firmly in place, which contributes to the high retention capacity of the Si@CS electrode. When PAAA was grafted onto CS, the CS-*g*-PAAA binder further suppressed the volumetric expansion of Si, because this unique combination forms a stable 3D network with Si particles. This enhanced the CE of the Si@CS-*g*-PAAA electrodes by minimizing the Si isolation which occurs with the volumetric expansion of Si, and this helped to maintain the electrode's capacity over numerous cycles.^52^

Secondly, the direct contact between the conductive PAAA and Si particles not only facilitated electrochemical reactions but also maintained SEI, thus achieving low resistance and low polarization. The electrode characteristics, however, can vary depending on the ratio of CS to PAAA, and it was found that cell performance may deteriorate with an excess amount of PAAA due to poor adhesion of the corresponding binder.

Overall, the Si@CS-*g*-PAAA-8 : 1 electrode displayed the highest CE and retention capacity because its optimally balanced ratio of CS to PAAA resulted in optimized adhesion strength and electrical conductivity.

**Fig. 6:**
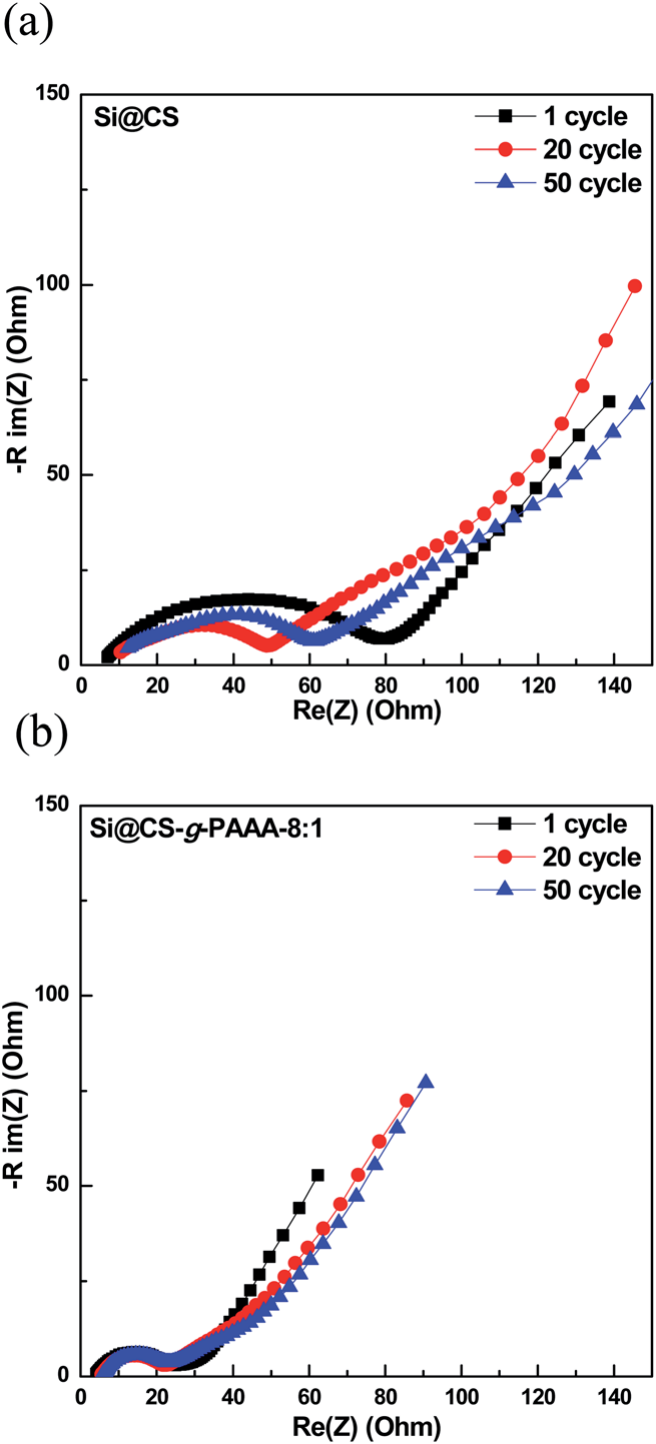
Electrochemical impedance spectroscopy graphs of (a) Si@CS after 1 cycle, 20 cycles and 50 cycles, and (b) Si@CS-*g*-PAAA-8 : 1 binder electrode after 1 cycle, 20 cycles and 50 cycles.

The Si@CS-*g*-PAAA-8 : 1 electrode, which had the best cell performance, was then subjected to charge/discharge cycling at high current, of 4.2 A g^−1^ (1C), and the EIS measurements taken during the process were compared to those of the pristine Si@CS electrode (Fig. 6a and b). The resistance of the pristine Si@CS electrode without PAAA was higher than that of the Si@CS-*g*-PAAA-8 : 1 electrode (Fig. 6a). As mentioned before, the grafting of conductive PAAA lowered the resistance of the SEI layer on the Si electrode surface through the direct contact between this binder and Si, and thus, the Si@CS-*g*-PAAA-8 : 1 electrode showed a lower resistance and also maintained that low resistance over multiple cycles (Fig. 6b).

**Fig. 7:**
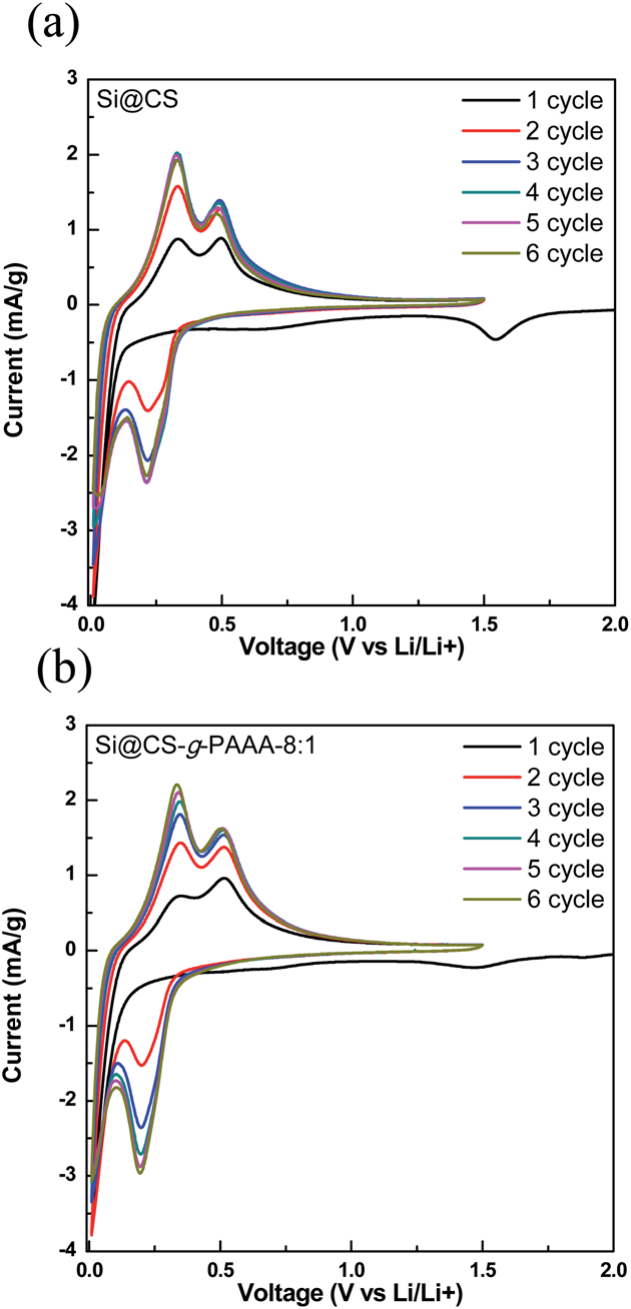
Cyclic voltammetry profiles of (a) Si@CS and (b) Si@CS-*g*-PAAA-8 : 1 at 1–6 cycles.

Next, cyclic voltammetry (CV) was performed to examine the reversibility of electrochemical reactions on the Si@CS-*g*-PAAA-8 : 1 electrode, and the results were compared to that of the Si@CS electrode (Fig. 7a and b). Peaks indicating irreversible SEI formation were observed in the range of 1.0 V to 0.5 V for both the Si@CS and Si@CS-*g*-PAAA-8 : 1 electrodes. The absence of such peaks in the second cycle, however, suggests that side reactions did not occur during the following charge/discharge cycles.

Taking advantage of the mechanical and electrochemical properties of the electrodes made with the CS-*g*-PAAA binders, we further prepared an electrode using the ECS-*g*-PAAA-8 : 1 binder, which was obtained by grafting PAAA onto chitosan extracted from crab shells. The cycle data was compared with the results from the commercially available chitosan-*grafted*-PAAA (Si@CS-*g*-PAAA-8 : 1) electrode (Fig. 5d). The Si@ECS-*g*-PAAA-8 : 1 electrode showed an initial capacity of 3057.3 mA h g^−1^, ICE of 75.7%, and a capacity of 1408.8 mA h g^−1^ after 300 cycles, which translates to an excellent capacity retention of 51.4% (Fig. 5d).

This is a significant improvement over past experiments with Si anode binders prepared using other natural substances, and is attributed to the stable 3D structure formed between the PAAA and the CS functional groups, as well as the electrical conductivity of this binder.

The Si@ECS-*g*-PAAA-8 : 1 electrode synthesized using chitosan extracted from crab shells showed somewhat better performance than the Si@CS-*g*-PAAA-8 : 1 electrode, which had the same ratio of CS to PAAA, because there were slightly higher amounts of amines and amides groups in the extracted chitosan than in the commercially available chitosan (see above).

### 3.4. Morphological analysis of the Si@CS-*g*-PAAA electrodes

The morphologies of the Si@CS-*g*-PAAA-8 : 1 electrodes were investigated using scanning electron microscopy (SEM) images and EDS mapping of each electrode. The results were then compared to those of a Si@CS electrode (Fig. S4† and 8).

**Fig. 8:**
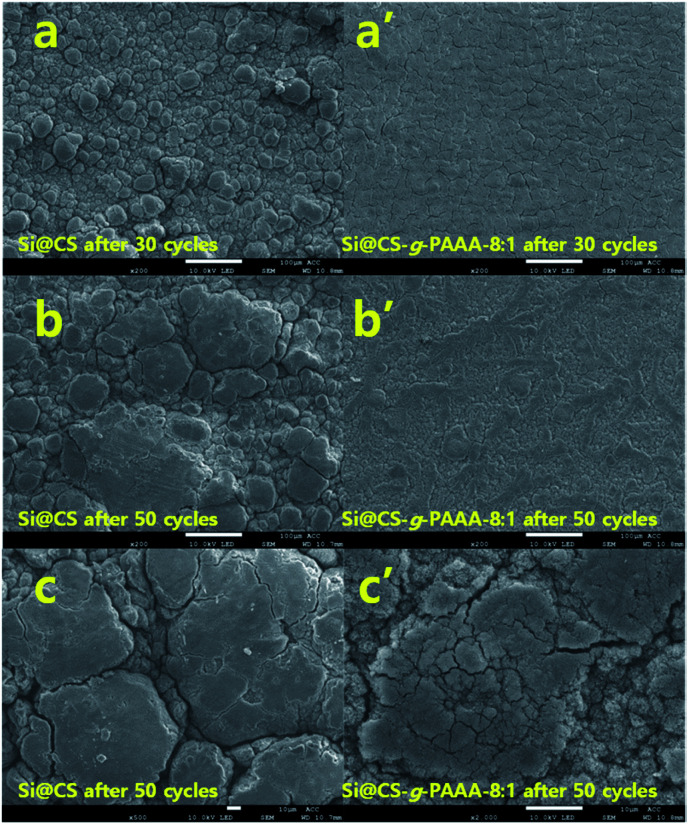
SEM images of (a) Si@CS and (a′) Si@CS-*g*-PAAA-8 : 1 after 30 cycles; (b) Si@CS and (b′) Si@CS-*g*-PAAA-8 : 1 after 50 cycles; (c) high magnification images of (c) Si@CS and (c′) Si@CS-*g*-PAAA-8 : 1 after 50 cycles.

Firstly, the EDS mapping of both electrodes before cycling revealed that the elements were less uniformly dispersed in the Si@CS electrode than in the Si@CS-*g*-PAAA-8 : 1 (Fig. S4†). This was due to aggregation with the Si particles in the Si@CS electrode, resulting from strong interactions within the molecules of the hydroxyl (OH) and amine (NH_2_) groups in chitosan, as well as strong interactions with Si–OH. Moreover, the weak interaction of CS with hydrophobic conductive materials such as Super P was ascribed to the non-uniform dispersion of the carbon element (Fig. S4a†).

This effect weakened with the incorporation of PAAA. Unlike the Si@CS electrode, aggregation was not observed in the Si@CS-*g*-PAAA-8 : 1 electrode (Fig. S4b†). However, the Si@CS-*g*-PAAA-4 : 1 electrode, having further PAAA content, did not uniformly disperse the silicon particles due to its very low adhesion to Si as confirmed from the adhesion test (Fig. S4c†).

Secondly, SEM analysis of both electrodes after 30 cycles revealed that the Si@CS-*g*-PAAA-8 : 1 electrode showed more uniform surface than that of the Si@CS electrode (Fig. 8a and a′). In addition, although the Si@CS-*g*-PAAA-8 : 1 binder had a lower adhesive strength than Si@CS, the cracking behavior between these two electrodes were almost similar. It was, therefore, concluded that the introduction of CS suppressed isolation of the Si from the Cu current collector. At the same time, similar cracks formed due to the strong adhesion of the CS binder in the Si@CS electrode. Aggregation was, however, widespread in the images of the Si@CS electrode, and this is consistent with it being a non-uniform electrode.

Finally, the morphologies of both electrodes were further compared after 50 cycles, and it was found that the morphology of the Si@CS-*g*-PAAA-8 : 1 electrode was not very different than the same electrode after 30 cycles (Fig. 8b′ and c′). However, the surface of the Si@CS electrode was covered with a thicker SEI layer (Fig. 8b and c). The EDS mapping images of the cells after the cycle also showed that the Si in the CS-*g*-PAAA-8 : 1 electrode was more uniformly dispersed with less aggregation than the CS electrode (Fig. S5†). These results indicated that the CS-*g*-PAAA-8 : 1 electrode had less isolated Si during the cycles. It had also formed a uniform SEI layer because of the strong adhesion, in combination with the good connection between this binder and Si, due to the formation of the 3D structure between CS-*g*-PAAA and Si particles. These results are in good agreement with the results for coulombic efficiency.

### 3.5. Solubility test of the binder

Lastly, a solubility test was conducted for the CS and CS-*g*-PAAA-8 : 1 binders. Because of its low solubility in 1 M acetic acid, chitosan had high viscosity and did not form a homogeneous solution, whereas CS-*g*-PAAA-8 : 1 was more soluble and thus produced a homogeneous solution (Fig. S6†). The CS binder, when used alone, has poor solubility in acetic acid, and the amount of solvent must be greater than the CS-*g*-PAAA-8 : 1 binder for complete dissolution. This limits its practical application, because using more solvent makes it difficult to adjust electrode viscosity, resulting in the formation of non-uniform electrodes. This was confirmed from the SEM EDS mapping results (Fig. S4†).

On the other hand, by reducing the amount of chitosan relative to the weight of the binder, the CS-*g*-PAAA-8 : 1 binder was able to form better electrodes with less solvent (1 M acetic acid). This resolved the problem of excess solvent and also created uniform electrodes. As such, the proposed binder is expected to have significant advantages in the manufacturing of electrodes for practical application.

## 4. Conclusion

We synthesized CS-*g*-PAAA with various CS to PAAA compositions by grafting conductive PAAA onto chitosan, and characterized their structures using FT-IR and TGA. The amount of PAAA to chitosan was varied to assess the effect on the physical and electrical properties of the cells made of CS-*g*-PAAA.

In addition to being electrically conducting, the PAAA structure contains carboxylic acid functional groups which bind to silicon particles, along with the chitosan functional groups, to form a stable 3D network. This results in high Si adhesion. The electrode with the best balance of conductivity and adhesion was the electrode prepared from CS-*g*-PAAA-8 : 1 with an optimal composition of CS to PAAA of 8 : 1. It exhibited a high initial capacity of 2785.6 mA h g^−1^, and maintained a high capacity of 1301.0 mA h g^−1^ after 300 cycles, a retention of 58.5%.

Furthermore, the Si@CS-*g*-PAAA-8 : 1 electrode showed more uniform surface than that of the Si@CS electrode, and the cracking behavior between these two electrodes were almost similar, suggesting that the introduction of CS suppressed isolation of the Si from the Cu current collector.

Chitosan was also directly extracted from crab shells, and an Si@ECS-*g*-PAAA electrode was fabricated by grafting PAAA onto the extracted chitosan (ECS). This electrode recorded an initial capacity of 3057.3 mA h g^−1^, and maintained a high capacity of 1408.8 mA h g^−1^ with 51.4% retention after 300 cycles.

The cell characteristics of our binders were compared with several other binders reported in the literature (Table S3†). As expected, the higher the loading level at the same current density, the lower the cell performances were obtained, and they are also greatly affected by the current density. Considering these, our CS-*g*-PAAA binder electrodes showed excellent cell performance even at high mass loading and high current density of 2 A g^−1^ or more.

This study developed a polymeric binder for Si anodes with outstanding cell properties, ease of fabrication, and high water solubility, by grafting a conductive PAAA onto chitosan, an abundant natural material. The study opens new possibilities for the development of affordable, eco-friendly binders for Si anodes in lithium ion batteries.

## Conflicts of interest

The authors declare no competing financial interests.

## Supplementary Material

RA-010-C9RA10990K-s001
