# Supplementary material for: Chitosan-grafted-poly(aniline-co-anthranilic acid) as a water soluble binder to form 3D structures for Si anodes
Source: RSC Adv. 2020 Feb 19;10(13):7643–53. doi: 10.1039/c9ra10990k (PMC9049896; doi:10.1039/c9ra10990k)
Supplement: RA-010-C9RA10990K-s001 [file RA-010-C9RA10990K-s001.pdf]

Electronic Supplementary Material (ESI) for RSC Advances.
This journal is © The Royal Society of Chemistry 2020

Electronic Supplementary Information for

Chitosan-*grafted*-poly(aniline-*co*-anthranilic acid) as a water soluble binder to form 3D structures for Si anodes

Eunsoo Kim,[a,b†] Rajeev K.K.,[a,b†] Jaebin Nam,[a,b] Junyoung Mun,[c] and Tae-Hyun Kim[a,b*]

[a]Organic Material Synthesis Lab. Department of Chemistry, Incheon National University, 119 Academy-ro, Songdo-dong, Yeonsu-gu, Incheon 22012, Korea.

[b]Research Institute of Basic Sciences, Incheon National University.

[c]Department of Energy and Chemical Engineering, Incheon National University.

[†] These authors contributed equally to this work.

Corresponding Author

[*]Tel: +82-32-835-8232; Fax: +82-32-835-0762; E-mail: tkim@inu.ac.kr (T.-H. Kim)

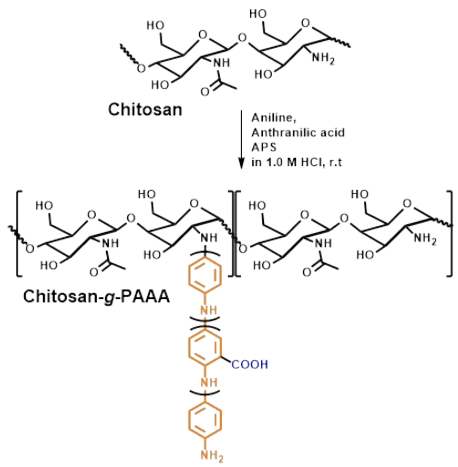

Scheme S1. Synthetic procedure for the chitosan-*grafted*-PAAA (CS-*g*-PAAA).

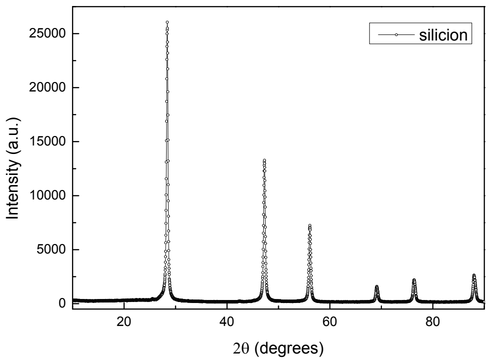

Figure S1. XRD pattern of silicon powder used.

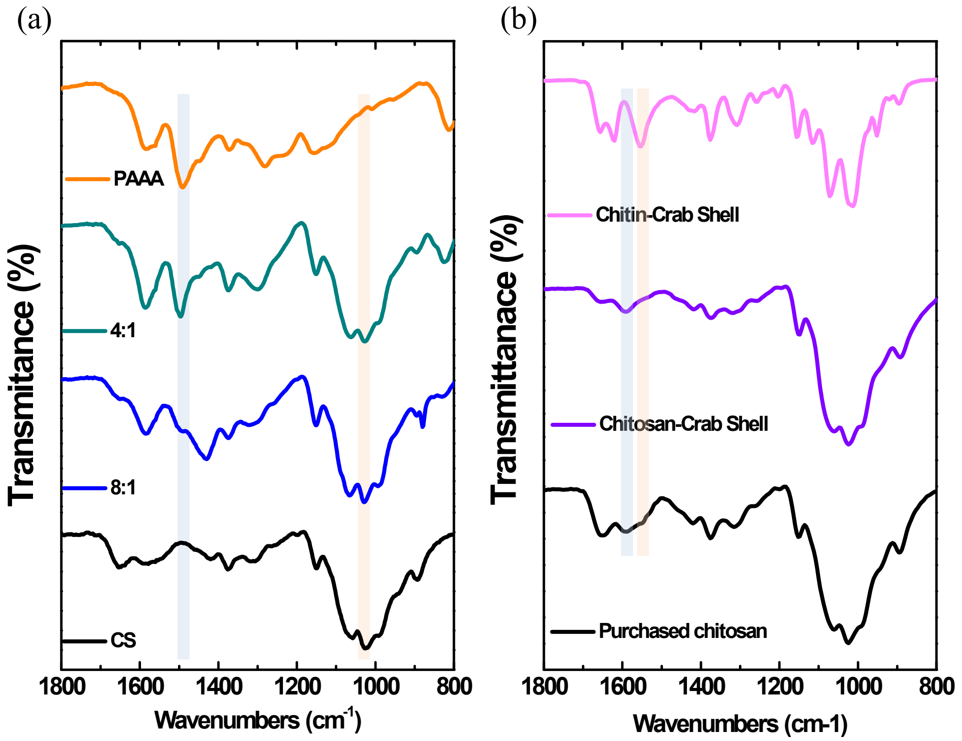

Figure S2. IR spectra of (a) CS (black line), CS-*g*-PAAA-8:1 (blue line), CS-*g*-PAAA-4:1 (dark cyan line) series and PAAA (orange line) and (b) commercially available CS (black line), CS from crab shell (violet line) and chitin extracted from crab shell (pink line).

Table S1. Main peaks of the IR spectra of CS, CS-g-PAAA and PAAA.

Assignment	Wavenumber (cm^{-1})		
	Chitosan	CS-*g*-PAAA	PAAA
Amide C=O stretch	1654	1652	-
Amide N-H bend	1592	1584-1600	-
Quinoid C=C stretch	-		1584
Benzenoid C=C stretch	-	1492-1506	1492
Glycosidic C-O-C stretch	1150	1140-1150	-
Aromatic C-H in-plane bend	-		1140
Carbonyl C-O stretch	1058, 1026	1058, 1026	-

Table S2. TGA data (remaining mass % and degradation %) of PAAA and CS-*g*-PAAA series and HCl treated chitosan.

Binder	Remaining mass %			180 ℃ - 320 ℃Degradation%
	180 ℃	320 ℃	700℃	
PAAA	97.21	90.13	44.67	7.283
CS-*g*-PAAA-4:1	92.85	56.97	36.35	35.41
CS-*g*-PAAA-8:1	92.54	51.78	34.45	44.05
HCl treated CS	90.10	46.75	28.05	48.11

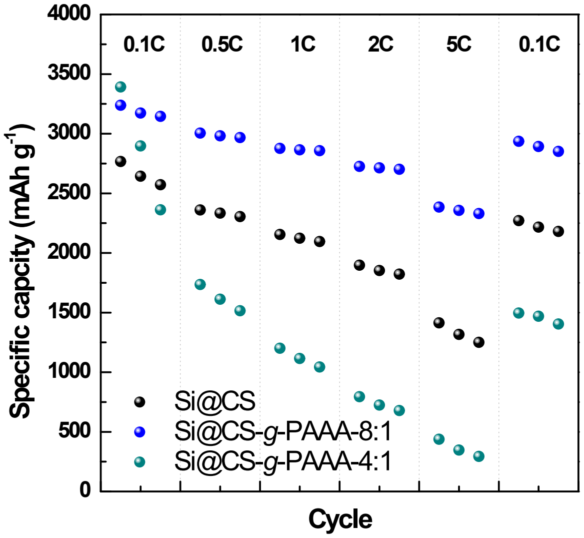

Figure S3. Rate capabilities of Si@CS, Si@CS-*g*-PAAA-8:1 and Si@CS-*g*-PAAA-4:1 electrodes at current densities varying between 0.1 C and 5 C.

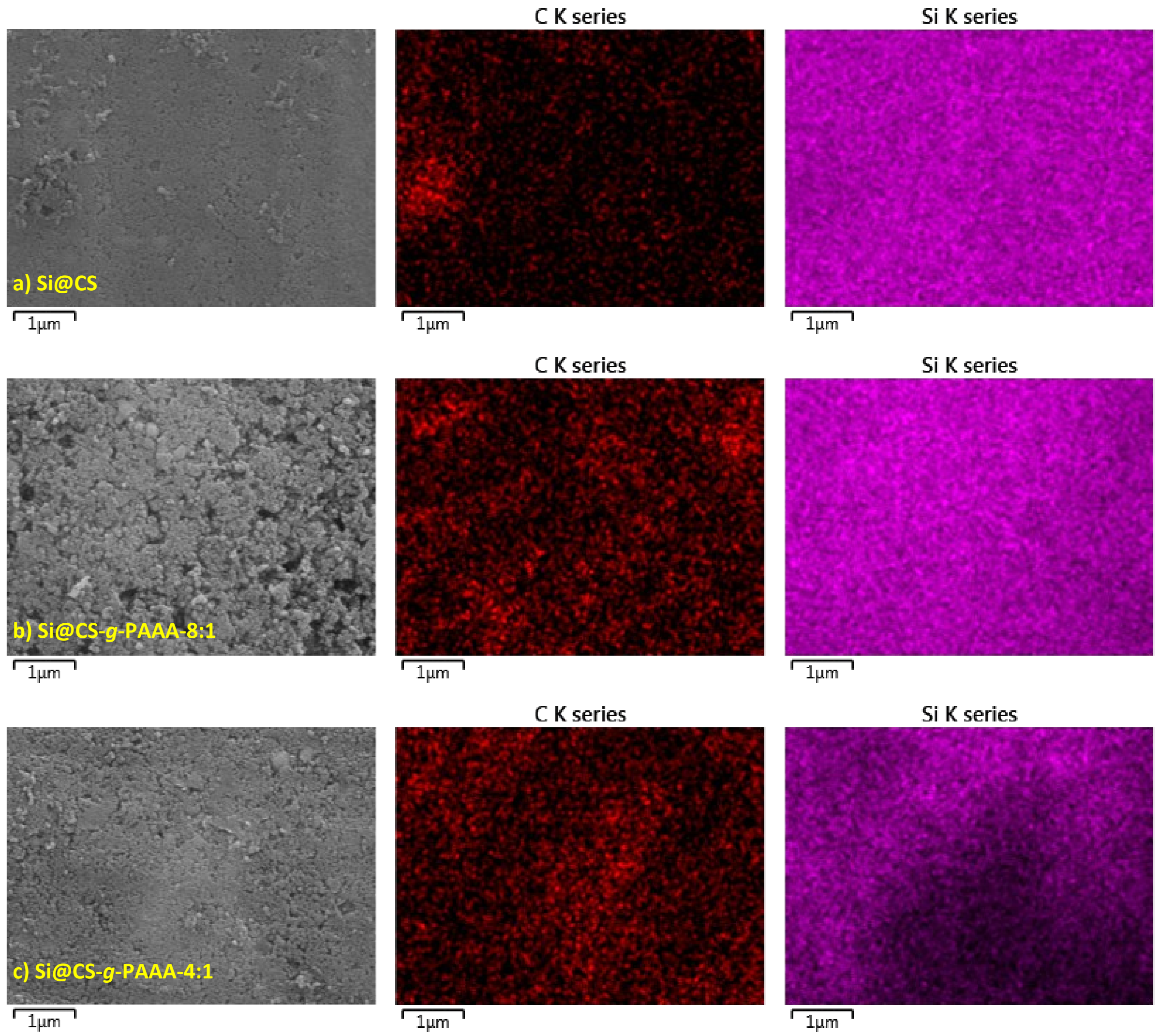

Figure S4. SEM-EDS mapping images of (a)Si@CS, (b)Si@CS-*g*-PAAA-8:1 and (c) (b)Si@CS-*g*-PAAA-4:1 electrodes.

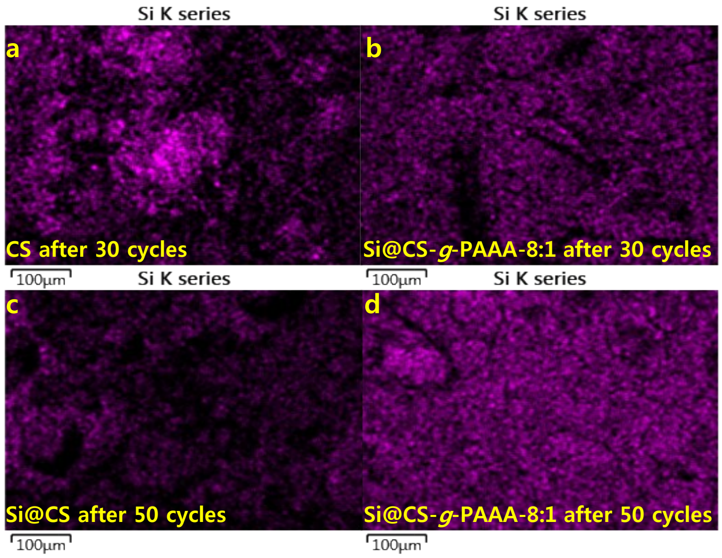

Figure S5. SEM-EDS mapping images of (a) Si@CS and (b) Si@CS-g-PAAA-8:1 after 30 cycles; (c) Si@CS and (d) Si@CS-*g*-PAAA-8:1 after 50 cycles.

(a) (b)

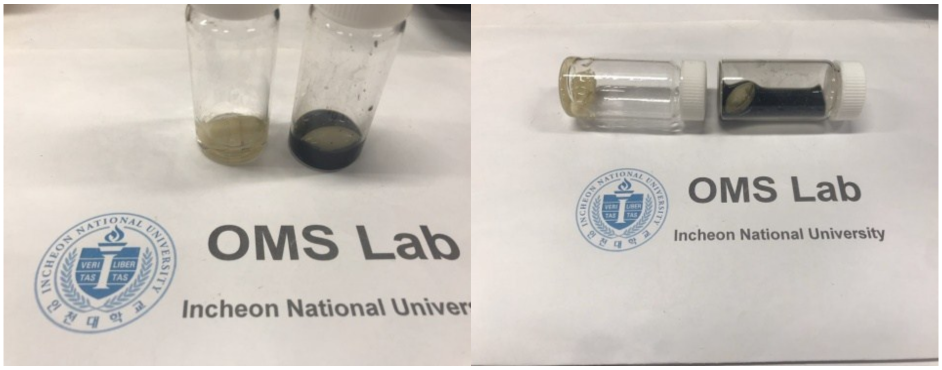

Figure S6. Solubility test results for (a) CS (left) and CS-*g*-PAAA 8:1 (right) binders in 1 M CH_3COOH before tilting and (b) after tilting. After tilting the parent solids sticking to the vial are visible.

Table S3. Comparison of the electrochemical performance for the Si electrodes fabricated by our novel binders with other representative binders.

Binder	Electrode mass loading	Current density	Electrode performance	Ref.
**CS-*g*-PAAA-8:1**	**0.67 mg cm^{-2}**	**2.1 A g^{-1}**	**1546 mAh g^{-1} @ 100th cycle**<br>**1301 mAh g^{-1} @ 300th cycle**	**This work**
PDA-PAA-PEO	0.4 mg cm^{-2}	2.1 A g^{-1}	1597 mAh g^{-1} @ 200th cycle	1
Na-CMC	unknown	2.1 A g^{-1}	1000 mAh g^{-1} @ 100th cycle	2
PAA-PEGPBI	1.0~1.3 mg cm^{-2}	2.1 A g^{-1}	1221 mAh g^{-1} @ 50th cycle.	3
PVA-PEI	3~4.5 mg cm^{-2}	1 A g^{-1}	1063 mAh g^{-1} @ 300th cycle	4
PVTES-NaPAA	0.5 mg cm^{-2}	840 mA g^{-1}	2077 mAh g^{-1} @ 100th cycle	5
NaPAA-*g*-CMC	0.45 mg cm^{-2} for Si	840 mA g^{-1}	1816 mAh g^{-1} @ 100th cycle	6
PAL-NaPAA	0.7 mg cm^{-2}	840 mA g^{-1}	1914 mAh g^{-1} @ 100th cycle	7
CS-CG+GA	0.4 mg cm^{-2}	420 mAh g^{-1}	2345 mAh g^{-1} @ 100th cycle	8
PAA/PANI IPN	1 mg cm^{-2}	420 mAh g^{-1}	2205 mAh g^{-1} @ 300th cycle	9
PEDOT:PSS/CMC	~1mg cm^{-2} for Si	420 mAh g^{-1}	1834 mAh g^{-1} @ 100th cycle	10
PVA-*g*-PAA	0.5-0.6 mg cm^{-2}	400mAh	1315 mAh g^{-1} @ 1000th cycle	11
PEEM	0.2 mg cm^{-2} for Si	375 mA g^{-1}	3000 mAh g^{-1} @ 50th cycle	12
PPQ	0.8 mg cm^{-2}	358 mA g^{-1}	2826 mAh g^{-1} @ 50th cycle	13
PANi	0.3~0.4 mg cm^{-2}	300 mA g^{-1}	2100 mAh g^{-1} @ 70th cycle	14

References

1 L. Lü, H. Lou, Y. Xiao, G. Zhang, C. Wang and Y. Deng, *RSC Adv.*, 2018, **8**, 4604-4609.
2 N. Ding, J. Xu, Y. Yao, G. Wegner, I. Lieberwirth and C. Chen, *J. Power Sources*, 2009, **192**, 644-651.
3 S. Lim, K. Lee, I. Shin, A. Tron, J. Mun, T. Yim and T. H. Kim, *J. Power Sources*, 2017, **360**, 585-592.
4 Z. Liu, S. Han, C. Xu, Y. Luo, N. Peng, C. Qin, M. Zhou, W. Wang, L. Chen and S. Okada, *RSC Adv.*, 2016, **6**, 68371-68378.
5 L. Wei, C. Chen, Z. Hou and H. Wei, *Sci. Rep.*, 2016, **6**, 1-8.

6 C. Luo, L. Du, W. Wu, H. Xu, G. Zhang, S. Li, C. Wang, Z. Lu and Y Deng, *ACS Sustainable Chem. Eng.*, 2018, **6**, 12621-12629.
7 X. Zeng, Y. Shi, Y. Zhang, R, Tang and L. Wei, *RSC Adv.*, 2018, **8**, 29230-29236.
8 X. Yu, H. Yang, H. Meng, Y. Sun, J. Zheng, D. Ma and X. Xu, *ACS Appl. Mater. Interfaces*, 2015, **7**, 15961-15967.
9 D. Shao, H. Zhong and L. Zhang, *ChemElectroChem*, 2014, **1**, 1679-1687.
10 P-F. Cao, G. Yang, B. Li, Y. Zhang, S. Zhao, S. Zhang, A. Erwin, Z. Zhang, A. P. Sokolov, J. Nanda and T. Saito, *ACS Energy Lett.*, 2019, **4**, 1171-1180.
11 J. He and L. Zhang, *J. Alloys Compd.*, 2018, **763**, 228-240.
12 M. Wu, X. Xiao, N. Vukmirovic, S. Xun, P. K. Das, X. Song, P. Olalde-Velasco, D. Wang, A. Z. Weber, L. W. Wang, V. S. Battaglia, W. Yang and G. Liu, *J. Am. Chem. Soc.*, 2013, **135**, 12048-12056.
13 S. M. Kim, M. H. Kim, S. Y. Choi, J. G. Lee, J. Jang, J. B. Lee, J. H. Ryu, S. S. Hwang, J. H. Park, K. Shin, Y. G. Kim and S. M. Oh, *Energy Environ. Sci.*, 2015, **8**, 1538-1543.
14 H. Wu, G. Yu, L. Pan, N. Liu, M. T. McDowell, Z. Bao and Y. Cui, *Nat. Commun.*, 2013, **4**, 1943-1946.
